# Microbial communities on eelgrass (*Zostera marina*) thriving in Tokyo Bay and the possible source of leaf-attached microbes

**DOI:** 10.3389/fmicb.2022.1102013

**Published:** 2023-01-06

**Authors:** Md Mehedi Iqbal, Masahiko Nishimura, Md. Nurul Haider, Susumu Yoshizawa

**Affiliations:** ^1^Atmosphere and Ocean Research Institute, The University of Tokyo, Kashiwa, Chiba, Japan; ^2^Department of Natural Environmental Studies, Graduate School of Frontier Sciences, The University of Tokyo, Kashiwa, Chiba, Japan; ^3^Faculty of Fisheries, Bangladesh Agricultural University, Mymensingh, Bangladesh

**Keywords:** *Zostera marina*, eelgrass microbiome, 16S rRNA gene, leaf age, suspended particles

## Abstract

*Zostera marina* (eelgrass) is classified as one of the marine angiosperms and is widely distributed throughout much of the Northern Hemisphere. The present study investigated the microbial community structure and diversity of *Z. marina* growing in Futtsu bathing water, Chiba prefecture, Japan. The purpose of this study was to provide new insight into the colonization of eelgrass leaves by microbial communities based on leaf age and to compare these communities to the root-rhizome of *Z. marina*, and the surrounding microenvironments (suspended particles, seawater, and sediment). The microbial composition of each sample was analyzed using 16S ribosomal gene amplicon sequencing. Each sample type was found to have a unique microbial community structure. Leaf-attached microbes changed in their composition depending on the relative age of the eelgrass leaf. Special attention was given to a potential microbial source of leaf-attached microbes. Microbial communities of marine particles looked more like those of eelgrass leaves than those of water samples. This finding suggests that leaf-attached microbes were derived from suspended particles, which could allow them to go back and forth between eelgrass leaves and the water column.

## 1. Introduction

Most terrestrial plants establish close relationships with microbes, ranging from mutualism to symbiosis (Kent and Triplett, 2002; Vorholt, 2012; Turner et al., 2013). Some plant-attached microbes are considered vital to the maintenance of host health and metabolic activity (Barott et al., 2011; Mishra and Mohanraju, 2018; Selvarajan et al., 2019). In the terrestrial ecosystem, rhizosphere (roots) microbial communities are generally composed of soil-derived microorganisms (Kent and Triplett, 2002). On the other hand, phyllosphere (leaf) microbial communities are mainly airborne, although their exact source is not well understood (Vorholt, 2012).

Seagrasses are the only paraphyletic group of angiosperms that have recolonized the marine environment. *Zostera marina* (eelgrass) is well known as a widespread seagrass species that generates ecological diversity and economically important ecosystems along coastlines throughout much of the Northern Hemisphere (Marba et al., 2006; Duffy et al., 2015; Fahimipour et al., 2017). Seagrass and other aquatic plants harbor various microorganisms in microenvironments, including the rhizosphere and phyllosphere. Their leaves stay underwater, whereas the root part is buried in the water-saturated sediment. Microorganisms loosely attached to them must be in and out of the plant ecosystem *via* water. Because of such a special environment, little is known about the interaction between aquatic plants and plant-attached microorganisms.

To date, one study has provided a detailed description of attached bacteria of aquatic angiosperms using denaturing gradient gel electrophoresis (DGGE: Crump and Koch, 2008). Another study identified epiphytic microbial communities on three seagrass species from the East African coast (Uku et al., 2007). Recently, some culture-independent surveys of seagrass epiphytic microbes have been published (Mejia et al., 2016; Ettinger et al., 2017; Fahimipour et al., 2017; Crump et al., 2018; Hurtado-McCormick et al., 2019, 2020; Ugarelli et al., 2019; Vogel et al., 2020; Iqbal et al., 2021; Rabbani et al., 2021). Most community analyses revealed that microbial communities varied in microbial composition with seagrass tissues: leaves and the root-rhizome (Mejia et al., 2016; Ettinger et al., 2017; Fahimipour et al., 2017; Crump et al., 2018; Ugarelli et al., 2019; Iqbal et al., 2021). Seagrass microbes have also been shown to differ between the root-rhizome and surrounding sediment (Cúcio et al., 2016; Fahimipour et al., 2017; Ettinger et al., 2017). Fahimipour et al. (2017) also found that leaf microorganisms were similar to underwater microorganisms. However, other research groups reported that there were large differences between leaf microbial communities and underwater communities (Mejia et al., 2016; Crump et al., 2018; Vogel et al., 2020). These studies focused on the microbial communities of seagrass leaves and did not differentiate among differently aged leaves. A bunch of eelgrasses is generally made up of leaves of different ages (Wium-Andersen and Borum, 1984; Törnblom and Søndergaard, 1999). Only one study, focused on leaf age and found that the microbial populations differed in abundance with increasing leaf age (Törnblom and Søndergaard, 1999).

Marine ecosystems involve seawater that contains suspended particles of various sizes. This adds another characteristic when comparing to terrestrial ecosystems. Aquatic bacteria are generally separated into two lifestyles: particle-associated (PA) and free-living (FL). In the marine environment, suspended particles are densely colonized by marine bacteria that can adhere easily to available surfaces (Iqbal et al., 2021). These particles may play a role in transferring adherent bacteria to the surface of other objects. It is presumed that bacteria move back and forth between plant bodies and the surrounding water through the process of particle formation, adhesion, and collapse (Iqbal et al., 2021).

The purpose of this study was to provide new insight into the colonization of eelgrass leaves by microbial communities based on leaf age and to compare these communities to the root-rhizome of *Z. marina*, and the surrounding microenvironment (i.e., PA and FL fraction of seawater and sediment). Each sample’s microbial communities were characterized by Illumina-based 16S rRNA gene amplicon sequencing. Here, the authors focused on the following questions: first, how are leaf microbial communities formed during the initial growth process of eelgrass leaves? Second, what are the major microbial groups thriving in *Z. marina* leaf and root-rhizome samples? Third, how do the microbial groups attached to the leaf surface change with leaf age?

## 2. Materials and methods

### 2.1. Site description and sample collection

**Figure 1:**
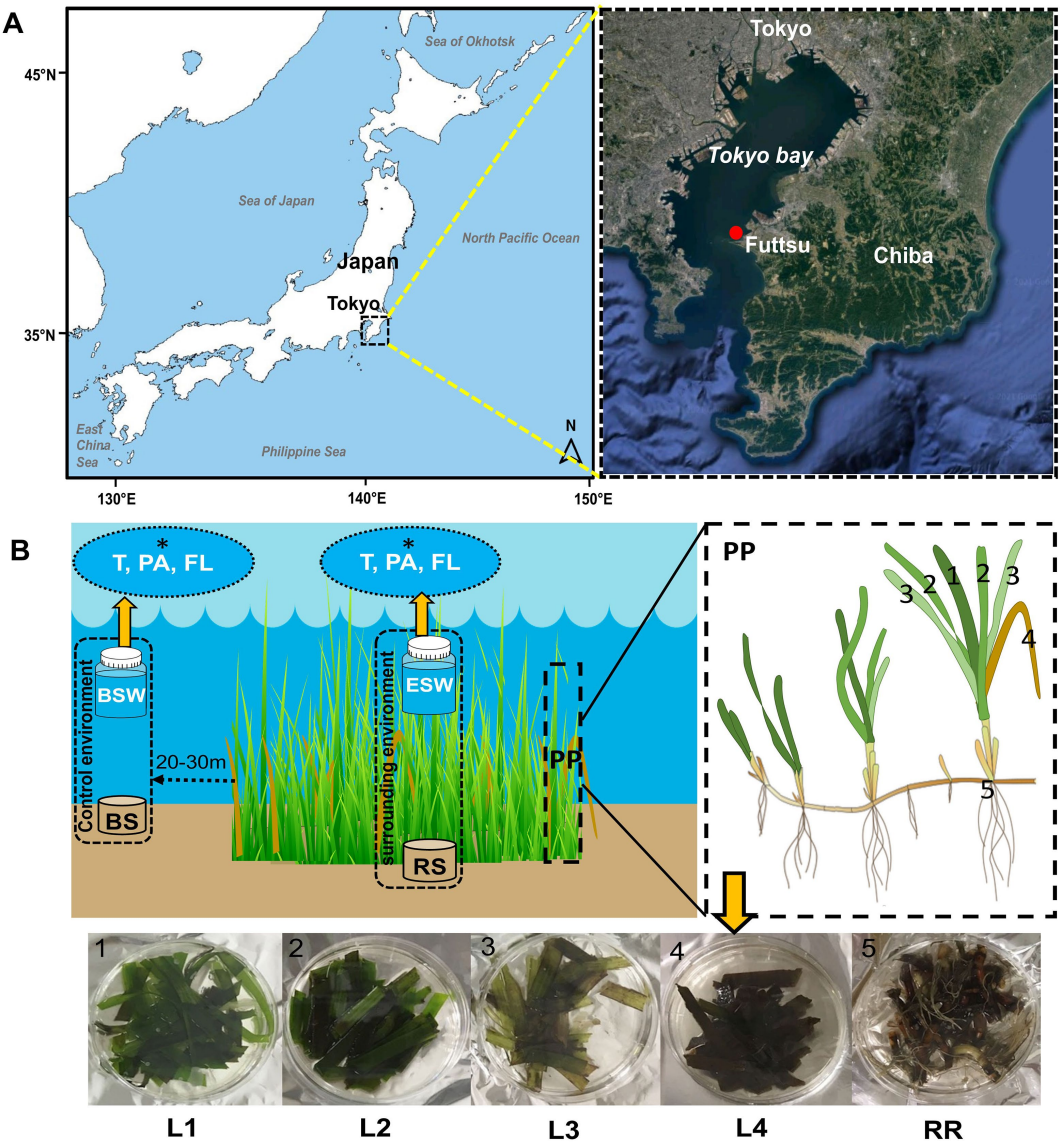
Location of the sampling areas and sampling strategy. **(A)** Samples were collected from *Z. marina* beds growing at Futtsu clam-digging beach, Chiba Prefecture, Japan, in July and September 2019. **(B)** At each sampling time, samples were collected from *Z. marina* plant parts (PP), such as the phyllosphere/leaves (1–4; L1, L2, L3, and L4), rooting zone (5; RR: root-rhizome), and surrounding environments (ESW, eelgrass surrounding water; RS, rhizosphere sediment) and control environment (BSW, bulk seawater; BS, bulk sediment). Within the phyllosphere sample, L1 is considered the youngest leaves, followed by older leaves L2 and L3, whereas L4 is the decayed leaves. Seawater samples were filtered for seawater microenvironment (i.e., T: total (PA + FL); PA, particle-associated; FL, free-living fraction; *white fonts).

Test samples were collected from eelgrass (*Z. marina*) beds growing at Futtsu clam-digging beach (35°18′56.71″N, 139°47′21.56″E and 35°18′53.39″N, 139°47′15.44″E; details shown in Table 1), Chiba Prefecture, Japan (Figure 1A). Currently, only three eelgrass beds are existed in the inner Tokyo Bay, the largest of which is located on the Futtsu tidal flat in the eastern Tokyo Bay (Yamakita et al., 2011). In previous studies (2015; unpublished), comparisons with other eelgrass bed (Ikuno-shima Is., Nanao Bay, and Mutsu Bay) around Japan, Futtsu showed a faster tidal flow. Guo and Yanagi (1996) also demonstrated strong residual currents in the middle of Tokyo Bay. In this area, some anthropogenic disturbance, such as raking for clams by local fisherman, was constantly observed (Yamakita et al., 2011).

**Table 1:** Location of sampling points and environmental variables of the seawater at each sampling date.

Sampling area	Sampling date	Sampling point	Location coordinates	Collected samples	Depth (m)	Temperature (°C)	Salinity (PSU)^b^	pH	DO (mg/L)
Futtsu, Chiba^a^	July 4, 2019	Inside	35°18′56.71″N, 139°47′21.56″E	Sediment, Seawater, plant part	1.0 ± 0.2	24.3 ± 0.1	31.6 ± 0.1	8.3 ± 0.4	10.1 ± 0.4
		Outside	35°18′51.75″N, 139°47′7.99″E	Sediment, Seawater	0.7 ± 0.2	24.0 ± 0.1	32.1 ± 0.1	8.1 ± 0.0	9.5 ± 0.3
	September 30, 2019	Inside	35°18′53.39″N, 139°47′15.44″E	Sediment, Seawater, plant part	1.1 ± 0.3	24.9 ± 0.1	32.0 ± 0.4	8.2 ± 0.0	10.1 ± 0.3

Temperature, salinity, pH, and dissolved oxygen (DO) were measured approximately 20 cm above the sediment. (a) Prefecture name, (b) PSU, Practical Salinity Unit. Values represent the mean of three replicates ± standard deviation.

Considering the eelgrass growing seasons, all samples were collected in summer (July 2019) and autumn (September 2019) during low tide (0.5–1.5 m water depth, Table 1). The test samples used for microbiome analysis and environmental measurement were collected simultaneously. Temperature, pH, salinity, and dissolved oxygen (DO) were measured at approximately 20 cm above the sediment using a Multiparameter Water Quality Checker (U series, HORIBA, JAPAN).

For microbial analyses, the test samples were recorded by various sample types, as shown in Table 2 and Figure 1B. Ten or more bunches of eelgrasses were pulled out at predetermined (10–20 cm) intervals by a gloved hand at each sampling time. These plant bodies were then gently washed with on-site water to remove sediment particles from the eelgrass surface. To remove loosely associated microbes and plankton, the eelgrass surface was rinsed with filtered and autoclaved seawater (Weidner et al., 1996; Burke et al., 2009; Iqbal et al., 2021). Then, the eelgrass bodies were cut into two parts: aboveground components (trunk and leaves) and belowground components (the root-rhizome) and placed into a sterile plastic bag.

**Table 2:** Overview of samples collected on each sampling date and seagrass host parameters.

Sampling date	Sample category	Sample type	Sample type (detail)	Total leaf blade sampled (*n*)	No. of sample used for microbiome analyses (*n*)	Blade length (cm)	Blade width (cm)	Bacterial abundance (cells cm^−2^ or ml^−1^ or g^−1^)
July 4, 2019	Plant parts (PP)	Leaves/phyllosphere	L1	12	8	75.5 ± 1.0	0.7 ± 0.1	(6.1 ± 0.6) × 10^6^
			L2	8	3	79.2 ± 1.3	0.7 ± 0.1	(8.5 ± 0.7) × 10^6^
			L3	8	3	80.7 ± 3.4	0.8 ± 0.1	(1.5 ± 0.0) × 10^7^
			L4	10	3	62.1 ± 1.3	0.6 ± 0.1	(8.1 ± 1.2) × 10^6^
		Rooting zone	Root-rhizome (RR)	–	3	–	–	(5.2 ± 0.0) × 10^7^
	Surrounding environments	Seawater	Eelgrass surrounding water (ESW): inside	–	_a_(3 × 3)	–	–	_b_(5.3 ± 0.4) × 10^6^
		Sediment	Rhizosphere sediment (RS): inside	–	3	–	–	_c_(1.1 ± 0.2) × 10^8^
	Control environments	Seawater	Bulk water (BSW): outside	–	_a_(3 × 3)	–	–	_b_(3.2 ± 1.0) × 10^6^
		Sediment	Bulk sediment (BS): outside	–	3	–	–	–
September 30, 2019	Plant parts (PP)	Leaves/phyllosphere	L1	8	3	61.2 ± 1.1	0.6 ± 0.1	(5.1 ± 0.1) × 10^6^
			L2	8	3	68.6 ± 2.0	0.7 ± 0.1	(6.2 ± 0.4) × 10^6^
			L3	8	3	70.0 ± 2.0	0.8 ± 0.1	(1.0 ± 0.1) × 10^7^
			L4	10	3	57.6 ± 4.9	0.6 ± 0.1	(1.1 ± 0.2) × 10^7^
		Rooting zone	Root-rhizome (RR)	–	3	–	–	(2.7 ± 0.3) × 10^7^
	Surrounding environments	Seawater	Eelgrass surrounding water (ESW): inside	–	_a_(3 × 3)	–	–	_b_(4.2 ± 0.5) × 10^6^
		Sediment	Rhizosphere sediment (RS): inside	–	3	–	–	_c_(2. 3 ± 0.1) × 10^8^

(a) Three (3) replicate samples of three (3) seawater microenvironments (i.e., total, particle-associated, and free-living fraction). (b) Bacterial abundance (cells ml^−1^) in a seawater sample. (c) Bacterial abundance (cells g^−1^) in sediment samples (values represent the mean of three replicates ± standard deviation). L1 (youngest), L2 (middle age), L3 (oldest), and L4 (decayed) leaves.

Sediment and water samples were collected from inside the eelgrass bed. Control samples were also collected from outside the eelgrass bed, which was 20–30 m from the edge of the eelgrass patch (Figure 1B). Eelgrass surrounding water (inside) samples were taken just above (0.1–0.5 m) each stub of collected grass. The water sample was collected in a sterile plastic bottle (1 l) and filtered through a 3.0 μm pore-size membrane filter attached to the polypropylene holder (ADVANTEC, Tokyo, Japan) and a 0.22 μm pore-size Sterivex filtering unit (Millipore, Massachusetts, United States) with a peristaltic pump to harvest particle-associated (PA) and free-living (FL) bacteria, respectively. Another bottle of water was filtered through a 0.22 μm pore-sized Sterivex filter unit directly for the total (T) community: (PA + FL). After filtration, a 3.0 μm pore-size filter was removed from the holder and then transferred into a 5 ml sterile tube. Rhizosphere sediment (inside) samples were taken from near the collected plant root with a plastic core sampler (sterile 50 ml plastic syringe), and the sediment cores were sectioned from the bottom surface, 5 cm each. Then, every core tube was sealed at both ends with a rubber stopper and immediately placed on ice. All the samples were transported to the laboratory on ice within 2 h of collection. In the laboratory, all the samples were frozen at −80°C until further analyses.

The relative age of the leaf blade was estimated from the order of emergence compared with those of other leaves, as described in a previous report (Törnblom and Søndergaard, 1999). Eelgrass grows by a continuous formation of new leaves elongating from the base and this growth pattern enables the determination of leaf age (Wium-Andersen and Borum, 1984; Törnblom and Søndergaard, 1999). In the laboratory, all leaves were cut off at the base and separated into three groups according to estimated age (Figure 1B): L1 (youngest), L2 (middle age), and L3 (oldest). Decayed leaves (withered leaves) were identified by visual inspection and separated from the stub of the collected grass (Figure 1B). They were also cut off at the base, stored and labeled L4 (decayed).

### 2.2. Enumeration of total bacterial abundance

Total bacterial abundance was determined using the direct counting method previously described by Porter and Feig (1980). Water samples were collected from each location (inside and outside of the seagrass bed) in triplicate. The water samples were fixed in formalin to achieve a 2% final concentration and kept refrigerated in the dark until enumeration by epifluorescent microscopy (Haider et al., 2018). Surface-attached microbes were detached from eelgrass parts (leaves and the root-rhizome) according to the method of Törnblom and Søndergaard (1999) with slight modification. Briefly, leaf and root-rhizome samples were cut into 1.0 cm segments (area 1.0 cm^2^) and then placed in a 9 ml volume of filter-sterilized seawater (0.22 μm Millipore filters). For sediment samples, a 1.0 g portion of sediment (wet weight) was placed into a 9 ml volume of filter-sterilized seawater (0.22 μm Millipore filters). Then, the samples were sonicated for 5 min (Branson Sonifier 250) and stirred vigorously using a vortex (Vortex-Genie 2, Scientific Industries, Inc., NY, United States) to separate attached bacteria from the plant surface and sediment particles. After that, the samples were fixed in formalin to achieve a 2% final concentration and kept refrigerated in the dark until enumeration. Finally, each sample was filtered (1 ml) through a 0.2 μm pore size IsoporeTM filter (Merck Millipore Ltd., Tullagreen, Carrigtwohill Co. Cork, Ireland). Sediment samples were serially diluted with sterile-filtered seawater to obtain efficient concentration for filtration. The IsoporeTM filter was stained with DAPI (4′,6-diamidino-2-phenylindole) according to the method of Porter and Feig (1980). Total bacterial numbers were enumerated on epifluorescence microscopy (Olympus BX-51, Olympus Opticals, Tokyo, Japan).

### 2.3. DNA extraction and sequencing

DNA was extracted from each sample with a DNeasy PowerSoil DNA extraction kit (Qiagen, Germany) according to the manufacturer’s instructions with some modifications. The DNA extraction was performed in triplicate for each sample type except L1 leaves (*n* = 8) collected in July (Table 2).

For the DNA extraction from eelgrass bodies (i.e., leaves and the root-rhizome tissues) and sediments, a 0.25 g portion of the sample (wet weight) was placed directly into an extraction tube without grinding according to the instructions of the DNeasy PowerSoil DNA extraction kit. For the DNA extraction from water-suspended particles, a 3.0 μm pore-size filter with trapped particles was cut into small pieces and then placed directly into another extraction tube in the same way. For the DNA extraction from the free-living and total (T) fraction, a Sterivex filter with trapped free-living/total (T) bacteria was manually excised and removed from the plastic housing of the filter unit. Then, the removed filter was transferred into another extraction tube. The extracted DNA was finally cleaned with a NucleoSpin gDNA Clean-up kit (MACHEREY-NAGEL GmbH & Co. KG, Neumann-Neander-Str., Düren, Germany) according to the manufacturer’s instructions and stored at-30°C until further use.

The hypervariable V4 region of the 16S rRNA gene was amplified by polymerase chain reaction (PCR) using the primers 515F (5′-GTGCCAGCMGCCGCGGTAA-3′) and 806R (5′-GGACTACHVGGGTWTCTAAT-3′; Caporaso et al., 2012). The V4 regions were chosen for sequencing because they are capable to detect both bacterial and archaea taxons with high resolution (Parada et al., 2016; Wear et al., 2018) and show a fewer biases (Wear et al., 2018). PCRs were carried out in a 20 μl volume containing 1 μl (1 ng/μl) of DNA template, 13.20 μl of molecular biological grade double distilled water, 1.0 μl (10 μM) of each primer, 2 μl of 10× TaKaRa Ex Taq Buffer, 1.6 μl of TaKaRa dNTP mixture (2.5 mM each), and 0.2 μl of TaKaRa Ex Taq HS Polymerase (TaKaRa, Kusatsu, Shiga, Japan) in triplicate. Thermal cycling was carried out for 25 cycles under the following conditions: initial denaturation at 94°C for 2 min, denaturation at 94°C for 30 s, annealing at 55°C for 30 s, elongation at 72°C for 30 s and final elongation at 72°C for 5 min. After amplification, the presence of a PCR product was confirmed by agarose gel electrophoresis, and three PCR products of each sample were pooled together to reduce PCR bias. PCR products were further purified and normalized using Agencourt AMPure XP (Beckman Coulter Inc., Beverly, MA, United States). After purification, the second PCR was performed using primers with a tag sequence. The PCR products were purified again using Agencourt AMPure XP (Beckman Coulter Inc., Beverly, Massachusetts, United States) before sequencing on a MiSeq platform (Illumina Inc., San Diego, CA, United States).

### 2.4. Sequence data access

The sequence data from this study have been submitted to the DDBJ Sequence Read Archive (DRA) under the accession number DRA014809.

### 2.5. Sequence processing

Raw sequence data were processed using the program Quantitative Insights Into Microbial Ecology 2 (QIIME2, ver. 2020.2).^1^ First, the raw paired-end FASTQ reads were demultiplexed using the Fastq barcode splitter^2^ and imported into QIIME 2. Demultiplexed reads were quality filtered, denoised, chimera checked and dereplicated using a DADA2 denoise-paired plugin (Callahan et al., 2016) after inspection of quality profile plots of forward and reverse reads. The taxonomic affiliation was performed using the QIIME feature-classifier classify-sklearn on Greengenes v_13.8 (McDonald et al., 2012). Alignment was performed with the Mafft algorithm (Katoh and Standley, 2013) to build a phylogenetic tree using Fasttree software (Price et al., 2009) for its subsequent use in UniFrac (Lozupone et al., 2006) distance analysis. The amplicon sequence variant (ASV) table resulting from DADA2 was filtered to remove sequences classified as organisms other than Bacteria and Archaea (i.e., eukaryotes, chloroplasts, mitochondria, and unclassified sequences). After these filtering steps, the lowest number of sequences in one leaf sample dropped to 5,874. This reduction in the number of sequence reads can be largely attributed to the removal of chloroplast DNA from the leaf samples. To reduce bias caused by differences in sequencing depth during diversity estimation, we omitted one sample containing fewer than 23,000 sequences from our analyses, and the sequences were rarefied at 23,000 reads (2nd lowest read 23,094) using QIIME feature-table rarefy (Weiss et al., 2017).

### 2.6. Data visualization and statistical analyses

Community structure and composition analyses were performed by processing the ASV table in the R environment (R Core Team, 2020) with the Phyloseq (McMurdie and Holmes, 2013), microbiome (Lahti and Shetty, 2012–2019), vegan (Oksanen et al., 2020), and ggplot2 (Wickham, 2016) packages.

For alpha diversity, the authors calculated Chao1 (Chao et al., 2005) and Shannon indices (Shannon and Weaver, 1949) in R. To compare the microbial community composition among the samples including differently aged leaves, we measured weighted UniFrac (Lozupone et al., 2006, 2007) dissimilarities calculated in R using the phyloseq package. These dissimilarities were then plotted using principal coordinate analysis (PCoA) and nonmetric multidimensional scaling (NMDS). The PERMANOVA test was also performed *via* the “adonis” function of the “Vegan” package (Oksanen et al., 2020) with 999 permutations. Venn diagrams and heatmaps were generated using the ASV abundance table according to the web-based software Calypso version 8.84 (Zakrzewski et al., 2017).^3^ In addition, UpSet plots were generated using the R package UpSetR (version 1.4.0; Lex et al., 2014). To detect taxa with a significant differential abundance among the differently aged eelgrass leaves, linear discriminant analysis effect size (LEfSe) measurement (Segata et al., 2011) was created according to the web-based tool (Chong et al., 2020). For LEfSe, the Kruskal–Wallis test by rank was performed to detect the taxa with a significant abundance, followed by LDA to evaluate the effect size of each differentially abundant taxa. Values were considered significant at *p* < 0.05 for both statistical methods. Taxa with markedly increased effect size were defined as those with an LDA score (log10) > 3.

Kruskal–Wallis tests were performed in R to check the significance of differentiation among the groups for alpha diversity matrices, and then the Wilcoxon rank sum test was applied to test the pairwise group differences. To determine if environmental factors and host characteristics varied significantly among sampling dates, sampling points and/or the relative ages of seagrass leaves, the analysis of variances (ANOVA) was performed in R, after which Tukey’s *post hoc* honest significant difference (HSD) test (Tukey, 1953) was applied to test the pairwise group differences.

## 3. Results

### 3.1. Environmental variables and host plant characterization

The environmental parameters and sampling details are shown in Table 1. Water temperature varied significantly with changes in sampling point (ANOVA, *p* < 0.01) and date (ANOVA*, p* < 0.001). Salinity did not vary significantly with changes in date (ANOVA, *p* > 0.05); however, it varied significantly between sampling points (ANOVA, *p* < 0.05). In July 2019, outside waters had a higher average salinity (32.1 PSU) than inside waters (31.6 PSU). The pH and DO were relatively constant regardless of location and date (ANOVA, *p* > 0.05).

The length and width of leaf blades were found to be different (ANOVA, *p* > 0.001; Table 2) among eelgrass leaves of different ages (L1, L2, L3, etc.). The blade lengths varied not only with changes in leaf age but also with sampling date (ANOVA, *p* < 0.001; Table 2). However, the leaves did not significantly differ in leaf width between sampling dates (ANOVA, *p* = 0.4). L3 leaves reached the largest length and width values both in July (length, 80.7 cm and width, 0.8 cm) and September (length, 70 cm and width, 0.8 cm; Table 2). Meanwhile, decaying leaves (L4) were significantly smaller in length and width than leaves of other ages (Tukey’s HSD test, *p* < 0.01; Table 2).

Total cell counts, determined by DAPI staining and epifluorescence microscopy, are shown in Table 2. Overall, average total cell counts were high for root-rhizome samples (ranging between 2.7 × 10^7^ and 5.2 × 10^7^ cells cm^−2^) compared with leaf samples (ranging between 5.1 × 10^6^ and 1.5 × 10^7^ cells cm^−2^; Table 2). Regarding the total cell counts of the leaf surface, old leaves had more bacteria on the leaf surface than young leaves (Table 2). Total cell counts in eelgrass surrounding water (inside) were higher than those in bulk water (outside). The highest cell count was shown in the eelgrass surrounding water in July (5.3 × 10^6^ cells ml^−1^), whereas the lowest cell count was shown in bulk water (outside) in July (3.2 × 10^6^ cells ml^−1^; Table 2). In the rhizosphere sediment, the total number of bacteria was high compared with other sample types, ranging from 1.1 × 10^8^ to 2.3 × 10^8^ cells g^−1^ (Table 2).

### 3.2. Microbial community composition and diversity among eelgrass bodies and surrounding microenvironments

The microbial communities of test samples (i.e., leaves, the root-rhizome, surrounding water, rhizosphere sediment, bulk water, and sediment) were characterized by sequencing the V4 region of the 16S rRNA gene using the Illumina MiSeq platform.

**Figure 2:**
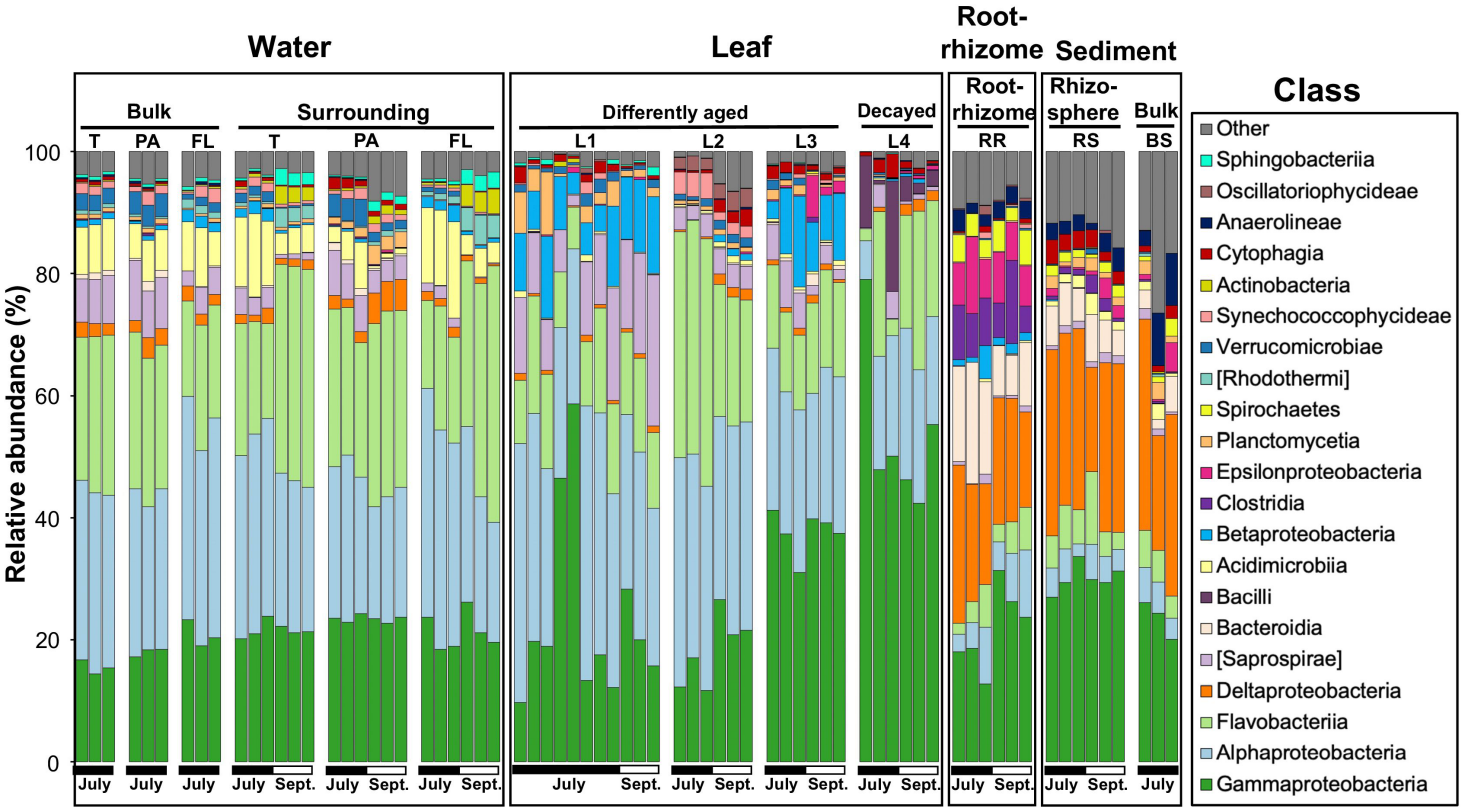
Microbial community composition of the eelgrass phyllosphere (differently aged and decayed leaves), rooting zone (the root-rhizome), surrounding environments (eelgrass surrounding water and rhizosphere sediment), and control environment (bulk water and sediment) at the class level in July and September. The class less than 2% was combined and referred to as “Other”. T, total (PA + FL); PA, particle-associated; FL, free-living; (L1–L3), differently aged leaves; L4, decayed leaves; RR, root-rhizome; RS, rhizosphere sediment; BS, bulk sediment.

A raw dataset consisted of a total of 8,591,113 sequences for all 71 samples. After quality filtering and removal of chimeras, chloroplasts, mitochondria, and unassigned sequences, a total of 4,954,679 high-quality sequences were retained for analysis (Supplementary Table 1). The average read number was 69,784 per test sample (min = 5,874; max = 101,948). In the high-quality sequences, the DADA procedure identified a total of 18,707 ASVs. To compare the diversity between the test samples, the number of sequences per sample was rarefied at 23,000 (2nd lowest) sequence reads (for more details, see Methods), and a total of 18,552 ASVs were obtained (Supplementary Table 1). Microbial communities associated with *Z. marina* (leaves and the root-rhizome), those of surrounding environments (i.e., eelgrass surrounding water and rhizosphere sediment), and control environments (bulk water and sediment) are shown at the class level in Figure 2. The leaf microbial composition is very distinct from that of the rhizosphere sediment and similar to that of the surrounding water (Figure 2 and Supplementary Figure 1). There seems to be a similarity in microbial composition between the microorganisms in the root-rhizome and those in the rhizosphere sediment (Figure 2 and Supplementary Figure 1). We also observed from Figure 2 that the microbes on leaves consisted mainly of the classes *Alphaproteobacteria, Gammaproteobacteria, Flavobacteriia,* [Saprospirae], *Betaproteobacteria,* and *Bacilli* (Firmicutes), whereas the root-rhizome microbiome was mainly made up of classes *Deltaproteobacteria, Gammaproteobacteria, Epsilonproteobacteria, Bacteroidia*, and *Clostridia* (Figure 2).

**Figure 3:**
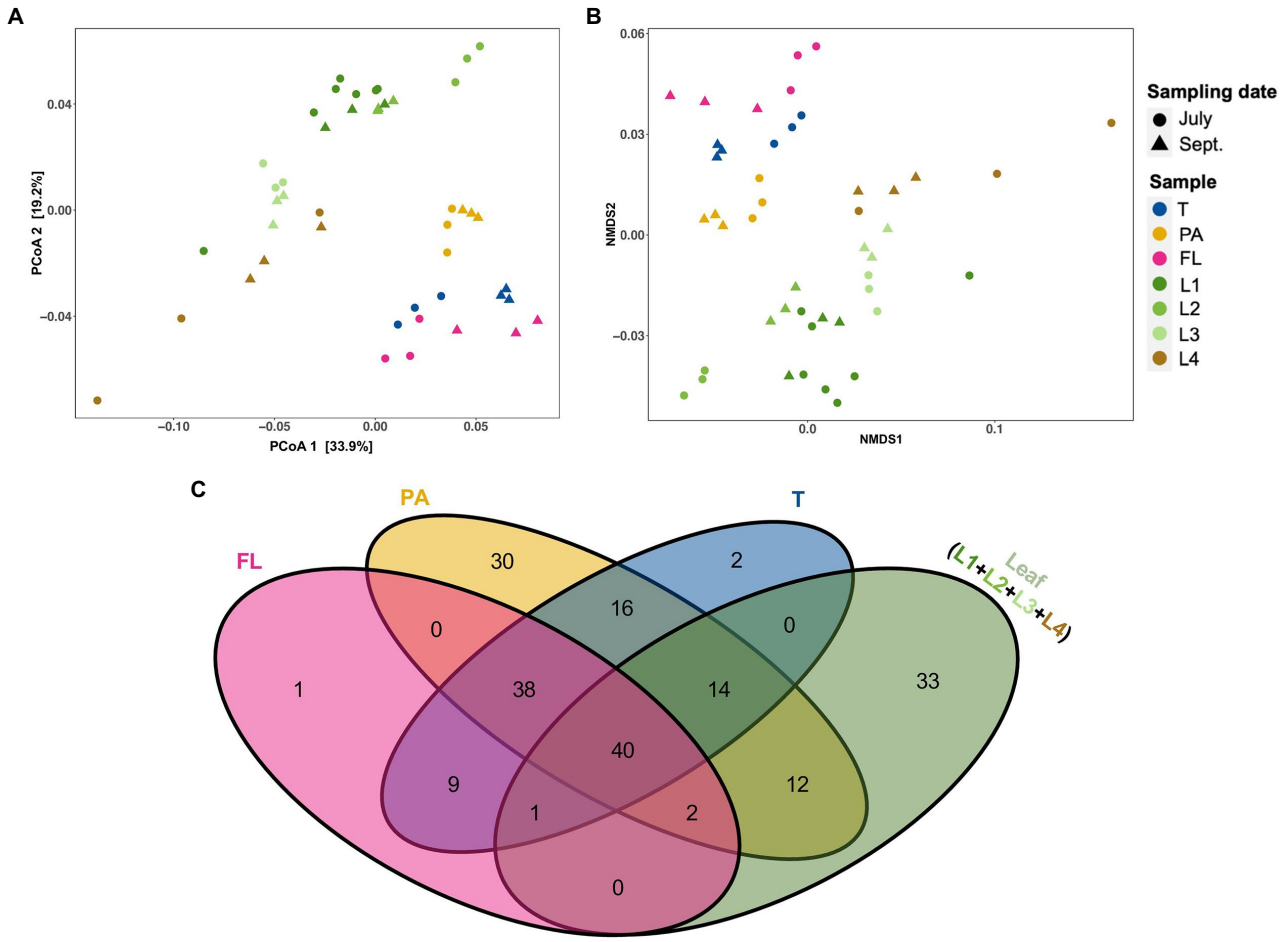
The relationship among eelgrass phyllosphere (differently aged and decayed leaves) and seawater microenvironment (i.e., T, PA, and FL fractions) microbiota. **(A)** Principal coordinates analysis (PCoA) and **(B)** nonmetric multidimensional scaling (NMDS) of microbial communities based on weighted UniFrac distances matrices showing the structural differences of microbial communities among phyllosphere and seawater microenvironments. Samples are colored by sample type (eelgrass surrounding water: T, PA, and FL fractions and leaves: L1–L4), with different shapes for sampling date (July, September). **(C)** Venn diagrams showing the unique and shared genera among the phyllosphere and surrounding water microenvironment. T, total (PA + FL); PA, particle-associated; FL, free-living; (L1–L3), differently aged leaves; L4, decayed leaves.

To recognize similarities in microbial composition between eelgrass leaves and surrounding microenvironments (i.e., PA, FL, and total fractions of water), both PCoA and NMDS analyses were performed based on weighted UniFrac distances (Figures 3A,B). Although leaf-attached microbial communities looked distinct from water-living communities, PA communities were plotted closer to the leaf communities than FL and total (T) (Figures 3A,B). In addition, to explore similarities between the eelgrass leaves and the surrounding seawater microenvironment (i.e., PA, FL, and T), shared and unique genera assigned per sample were visualized *via* a Venn diagram (Figure 3C). Among seawater microenvironments (i.e., PA, FL, and T), the PA fractions shared >65 genera with the leaves (i.e., L1, L2, L3, and L4), whereas the total (T) and FL fractions shared only 55 and 43 genera with the leaves, respectively (Figure 3C). Moreover, a total of 12 genera were exclusively shared between the PA fractions and the eelgrass leaves, while no genera were shared with the leaves and both total (T) and FL fractions (Figure 3C).

There were differences in alpha diversity between multiple test samples when analyzed either by Chao1 or Shannon’s diversity index (Kruskal–Wallis; *pChao1* < 0.001*, pShannon* < 0.001; Supplementary Figure 2). However, there was no significant difference in the alpha diversity between the two sampling dates (Kruskal–Wallis; *pChao1* = 0.345*, pShannon* = 0.459; data not shown here). Overall, diversity values were significantly higher (Wilcoxon rank sum test; *pChao1* < 0.05, *pShannon* < 0.05; Supplementary Figure 2) in the root-rhizome compared with the leaf samples. There was no significant difference in the alpha diversity between the root-rhizome and rhizosphere sediment (Wilcoxon rank sum test, *p* > 0.05; Supplementary Figure 2). In eelgrass surrounding water samples, PA fractions showed high diversity values (Wilcoxon rank sum test, *p* < 0.05; Supplementary Figure 2) compared with total (T) and FL fractions.

### 3.3. Variation in phyllosphere microbes with leaf aging

**Figure 4:**
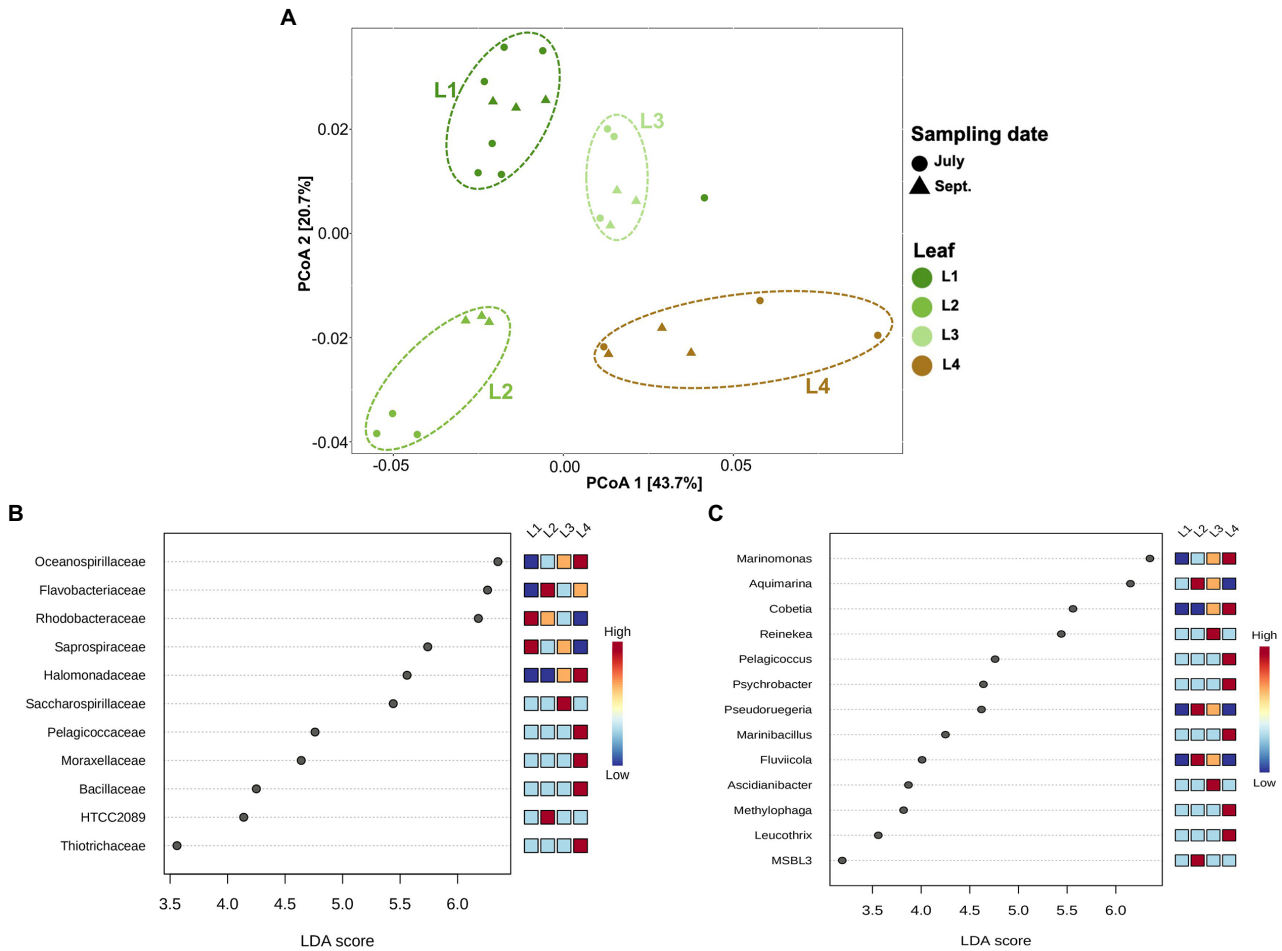
Principal coordinates analysis (PCoA) and LEfSe analysis among the phyllosphere **(A)** PCoA of microbial communities of the phyllosphere based on weighted UniFrac distance matrices. Samples are colored by leaf age (L1, L2, L3, etc.), with different shapes for the sampling date (July, September). **(B)** Linear discriminant analysis (LDA) effect size (LEfSe) analysis at the family level. **(C)** LEfSe analysis at the genus level. The differences were significant (*p* < 0.05) among classes (Kruskal–Wallis test). The threshold of the logarithmic LDA score was >3.0. (L1–L3): differently aged leaves; L4: decayed leaves.

In this experiment, eelgrass sampling was performed at different times in July and September, but there was not much difference in the microbial community on the eelgrass leaves depending on the sampling date (PERMANOVA; *R^2^ =* 0.06, *p* = 0.09; Figure 4A). Therefore, all eelgrass samples were grouped by leaf age (L1, L2, L3, and L4) regardless of collection date for subsequent comparison of the microbial community. While the community composition did not vary with the sampling date, it did vary with the relative leaf age (PERMANOVA; *R^2^* = 0.55, *p* < 0.001; Figure 4A).

The alpha diversity differed among eelgrass leaves of different ages (Kruskal–Wallis; *pChao1* = 0.002*, pShannon* = 0.004; Supplementary Figure 2). Older leaves (i.e., L2 and L3) showed high microbial diversity compared with young leaves (i.e., L1), whereas decaying leaves (i.e., L4) showed the lowest diversity value (Wilcoxon rank sum test; *pChao1* < 0.05*, pShannon* < 0.05; Supplementary Figure 2), except for Chao1 in L1.

Linear discriminant analysis (LDA) effect size (LefSe) was applied to characterize microbial communities, which had been found in differential abundance among the phyllosphere (Figures 4B,C). The families *Rhodobacteraceae* and *Saprospiraceae* were dominant on young leaves (L1 and L2). In contrast, the families *Oceanospirillaceae* and *Halomonadaceae* were found more on old and decaying leaves (L3 and L4; Figure 4B). The existence of microbial groups detected only from decayed leaves (L4) was also clarified; they consisted of four major groups, *Pelagicoccaceae*, *Moraxellaceae*, *Bacillaceae*, and *Thiotrichaceae* (Figure 4B). A look at the genus level identification (Figure 4C) reveals that the genera *Marinomonas* and *Cobetia* prefer to attach themselves to old and decaying leaves (L3 and L4) rather than to young leaves (L1 and L2; Figure 4C). The genera *Pelagicoccus, Psychrobacter, Marinibacillus, Methylophaga,* and *Leucothrix* were mainly enriched in the L4 leaves, and other genera (*Aquimarina, Pseudoruegeria, Fluviicola,* and MSBL3) were enriched in the L2 leaves. The genera *Reinekea* and *Ascidianibacter* were predominant on the L3 leaves (Figure 4C).

## 4. Discussion

### 4.1. Selection of 16S rRNA primers

PCR primers used to amplify 16S rRNA genes have changed substantially in recent years. For instance, many 16S rRNA gene primer pairs were designed for diversity studies of specific taxonomic groups such as the SAR11 clade (Apprill et al., 2015), and attempts have been made to develop a more universal primer pair that could cover close to the entire diversity of a natural microbial community (Caporaso et al., 2012; Gilbert et al., 2014). In this study, we focused on the comparative analysis of microbial communities to recognize the similarities in microbial composition between eelgrass bodies and surrounding environments, and thus, we selected a more universal primer sets (i.e., V4 primers; Caporaso et al., 2012) for the generation of amplicons. While a recent study by Wear et al. (2018) suggested that the selection of primer pairs did not affect the ecological interpretation of the results for aquatic planktonic communities, as the identified taxa had similar correlation patterns for all primer pairs. In addition, recent improvements of the 16S V4 primer sets have detected an increase amount of the SAR11 clade in the water column (Apprill et al., 2015; Parada et al., 2016). Although most of the previous studies did not report the SAR11 group that much abundance in the seagrass habitat (e.g., seagrass covering seawater: Fahimipour et al., 2017; Ugarelli et al., 2019; Iqbal et al., 2021). Additional studies need to be conducted to confirm whether the SAR11 clade presents at high abundance in the seagrass samples using a primer sets designed by Apprill et al. (2015).

### 4.2. Diversity of phyllosphere and rooting zone (the root-rhizome) microbial communities

The data presented here showed that the alpha-diversity of rooting zone microbes was significantly higher than that of phyllosphere microbes (Supplementary Figure 2), which is consistent with the results of previous studies on aquatic plants (Crump and Koch, 2008; Fahimipour et al., 2017; Hurtado-McCormick et al., 2019). Old leaves showed a great diversity of epiphytic microbes compared with young leaves, whereas decaying leaves showed the lowest microbial diversity (Supplementary Figure 2). Kohn et al. (2020) suggested that old leaves of *Posidonia oceanica* had a high microbial diversity. Bengtsson et al. (2012) reported that the diversity of microbial communities attached to the leaf surface increased with the leaf age of *Laminaria hyperborea*. A likely explanation for this observation is the new microbial groups are continuously being added to the community, whereas old groups persist (Bengtsson et al., 2012). In addition, due to the accumulation of small injuries on the leaf surface caused by mechanical forces or grazing make more varied surface environment on the older leaf (Bengtsson et al., 2012). This may lead to more niches for microbes becoming available over time, due to the more structurally varied habitat and access to different carbon sources excretion from the eelgrass leaf (Törnblom and Søndergaard, 1999), for example. While microbial diversity increased with leaf age, decayed leaves had the lowest diversity in this study suggesting that decayed leaves may have stimulated selective microbial augmentation on their surface and decomposing bacteria out competed the leaf microbiome. Microscopic studies revealed that the rooting zone (the root-rhizome) harbored high-abundance microbes compared with the phyllosphere (Table 2). In the phyllosphere, microbial biomass fluctuated between 1.1 × 10^6^ and 3.1 × 10^7^ cells cm^−2^, as has been previously reported (Newell, 1981; Kirchman et al., 1984; Törnblom and Søndergaard, 1999). Previous studies suggest that young leaves harbor fewer microbes on their surface than old leaves and that surface-attached microbes increase in number with advancing leaf age (Törnblom and Søndergaard, 1999; Hempel et al., 2008).

### 4.3. Community composition of phyllosphere and rooting zone (the root-rhizome) microbes

There were distinct communities associated with the phyllosphere and the below ground rooting zone (the root-rhizome; Figure 2 and Supplementary Figure 1). The leaf-community plots also gradually varied with leaf age (Figure 4A). The results are similar to previous studies in that the leaf-attached microbes were distinct in community structure from the rooting zone microbes in seagrass (Crump and Koch, 2008; Ettinger et al., 2017; Fahimipour et al., 2017; Crump et al., 2018; Ugarelli et al., 2019). However, to the best of our knowledge, this is the first report to show the relationship between eelgrass leaf age and the community structure of leaf-attached microbes. In the terrestrial environment, microbial colonization on a plant body differs from one individual to the next (Andrews and Harris, 2000). The foliar microbiological environments are related to various physiological states of plants, such as leaf age (Yang and Crowley, 2000). Regarding aquatic plants, it is known that the community structure of adherent microbes is subject to the organic compounds provided from the plant tissue (Ugarelli et al., 2019). For example, seagrasses nutritionally support foliar microbial communities (Kirchman et al., 1984; Törnblom and Søndergaard, 1999) by providing a carbon source (Moriarty et al., 1986; Williams et al., 2009). Moreover, another previous study reported that a certain type of eelgrass microbes synthesizes proteins as a result of advanced age, possibly due to excretion from the plant body (Törnblom and Søndergaard, 1999).

The root microbiome was mainly composed of the classes *Delta-, Epsilon-,* and *Gammaproteobacteria* and *Bacteroidia* (Figure 2). This is supported by recent studies of *Z. marina* plant microbiology, in which the authors found that Proteobacteria, especially the classes *Delta*, *Gamma*, and *Epsilonproteobacteria,* were predominant in *Z. marina* root samples (Jensen et al., 2007; Ettinger et al., 2017; Fahimipour et al., 2017; Crump et al., 2018). Microorganisms inhabiting seagrass roots are thought to play a major role in the cycling of the bioelements nitrogen, sulfur, and carbon (Lee and Dunton, 2000; Nielsen et al., 2001; Jones et al., 2003; Holmer et al., 2004; Cúcio et al., 2016; Kim et al., 2017; Crump et al., 2018).

The phyllospheric microenvironments were dominated mainly by the classes *Alphaproteobacteria*, *Gammaproteobacteria, Flavobacteriia*, and [Saprospirae] (Figure 2). However, *Alphaproteobacteria* and [Saprospirae] appeared most frequently on young leaves (Kruskal–Wallis; *p* < 0.001); *Gammaproteobacteria* became a major component group of old leaves (*p* < 0.001; Supplementary Figure 3). These findings agree with previous reports that the classes *Alpha-and Gammaproteobacteria* were predominant in the seagrass phyllosphere (Jiang et al., 2015; Mejia et al., 2016; Hurtado-McCormick et al., 2019). However, the results of this study make it clear that phyllospheric microbes vary in taxonomic composition with the aging of the leaf blade. Namely, the class *Alphaproteobacteria* (especially the family *Rhodobacteraceae*) is predominant on young leaves; however, the class *Gammaproteobacteria* (especially the families *Oceanospirillaceae* and *Halomonadaceae*) is predominant on old leaves (Figures 2, 4B). Over the past several years a dozen studies have been made on the relationship between the family *Rhodobacteraceae* and seagrass sample (Crump and Koch, 2008; Cúcio et al., 2016; Mejia et al., 2016; Ettinger et al., 2017; Rotini et al., 2017; Ugarelli et al., 2019). The family *Rhodobacteraceae* consists of chemo-organotrophs and photoheterotrophic bacteria, which are abundant and ubiquitous in coastal water. Some of them are known to produce antibacterial compounds (Dang et al., 2008). Also, some members of *Rhodobacteraceae* have been shown to have the nitrogen fixation ability (Palacios and Newton, 2005; Dang et al., 2008). The families *Oceanospirillaceae* (especially genera *Marinomonas*) and *Halomonadaceae* (especially genera *Cobetia*) were dominant in the biofilm on old and decaying leaves (L3 and L4; Figures 4B,C). The genus *Marinomonas* is involved in the degradation of lignin-derived compounds (Lu et al., 2020). The lignin is a major component of vascular plant tissue (Pollegioni et al., 2015), it also found in seagrasses (Opsahl and Benner, 1993; Klap et al., 2000; Pfeifer and Classen, 2020). The genus *Cobetia* has been isolated from green macroalgae *Ulva* sp., with some other species assimilating various carbon sources to produce polyhydroxyalkanoate (PHA; Gnaim et al., 2021). Previous studies of the eelgrass microbiome have focused on randomly selected leaf blades, regardless of leaf age (Ettinger et al., 2017; Fahimipour et al., 2017; Iqbal et al., 2021). This scientific paper is written with the aim of paying attention to a gradual change in microbial community structure during the aging process of eelgrass leaves. These results indicate that the major groups of phyllospheric microbes are always affected by eelgrass leaf age during the process of growth and death of eelgrass colonies.

### 4.4. A possible source of eelgrass leaf microbes

Fahimipour et al. (2017) suggested that some of the leaf microbial communities were of seawater origin. We also found that there was a high degree of microbial similarity between the microbial communities on eelgrass leaves and those in the water layer (Iqbal et al., 2021). Perhaps it is correct to say that the microbial communities on eelgrass leaves originate from the microbes in the water layer. Aquatic bacteria are generally separated into two lifestyles: particle-associated and free-living. Suspended particles in the ocean are densely colonized by marine bacteria. These particles play a key role in transferring adherent bacteria from one attachment surface to another. From the viewpoint of microbial community composition, suspended particles (PA) showed a higher degree of microbial similarity with the leaves (L1, L2, L3, and L4) than water samples (total: T and FL; Figure 3). Additionally, it revealed that the differently aged leaves (L1, L2, L3) had a higher degree of microbial similarity, whereas the decaying leaves (L4) had the least similarity with suspended particles (PA; Figure 2 and Supplementary Figures 1, 4). The results indicate that the suspended particles act as a means of transportation for microorganisms between the leaf samples and water samples (total: T and FL). Marine bacteria that easily adhere to suspended particles can migrate to the surface of eelgrass leaves through particle attachment and establish a unique ecological niche on the leaf surface. In our hypothesis, some marine bacteria may change their place of residence from marine particles to the foliar environment through the process of particle adhesion. Dead seagrass leaves are gradually fragmented and decomposed in the seagrass bed, during which the leaf material can be converted into suspended particles by microbial activity (Bach et al., 1986; Peduzzi and Herndl, 1991). We can explain the difference in microbial composition between the PA samples and the leaf samples in Figure 2 as follows: the young-leaf (L1 and L2) community and the old leaf (L3) community look much like the PA community, whereas the decaying-leaf (L4) community looks partially different (see also Supplementary Figure 1). This difference is thought to be because it takes much longer for fragmented dead leaves to become suspended particles than for the foliar microbial communities to be formed *via* the adhesion of particles. Moreover, another research paper suggesting a strong relationship between soil microorganisms and rhizosphere microorganisms has also been published (Kent and Triplett, 2002). Considering these facts comprehensively, it seems reasonable to propose that a probable source of leaf microbes can be particle-attached microbes present in the surrounding seawater, whereas the root-rhizome microbes may colonize from the surrounding sediment.

**Figure 5:**
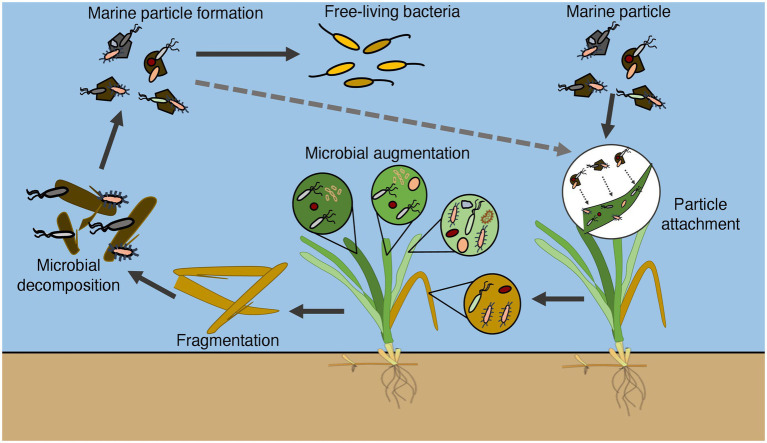
Conceptual diagram illustrating how marine bacteria are involved in the growth and decomposition process of eelgrass in Tokyo Bay.

A previous report showed that dead eelgrass leaves did not accumulate on the sediment surface but flowed out of the eelgrass bed by wind and the residual current in Tokyo Bay (Iqbal et al., 2021). The dead eelgrass leaves may be a unique carbon source of Tokyo Bay, which is rarely seen in other coastal waters with eelgrass beds. The results of this experiment revealed an ecological linkage between eelgrass microbial communities and marine particles. Combined with the results of a previous report, it was clarified how marine microbes are involved in the growth and decomposition process of eelgrass in Tokyo Bay (Figure 5).

## 5. Conclusion

The results of our experiment clearly showed that leaf microbial communities varied in accordance with the relative age of the eelgrass leaf. Microbial communities of marine particles looked more like those of eelgrass leaves than those of water samples (total: T and FL). On the other hand, a high similarity of microbial community structure was confirmed between sediment and rhizosphere samples. It was also observed in this experiment that leaf-attached microbes were derived from suspended particles, which could allow them to go back and forth between eelgrass leaves and the water column. Further study should be required to test whether the close relationship in microbial composition between particles and leaf microbes can be observed in a limited area, Futtsu, or over a wide spatial range.

## Data availability statement

The datasets presented in this study can be found in online repositories. The names of the repository/repositories and accession number(s) can be found at: https://www.ddbj.nig.ac.jp/, DRA014809.

## Author contributions

MI designed and performed the field and lab work, analyzed the data, and wrote the manuscript. MN supervised the study, performed the fieldwork, and provided manuscript editing. MH provided science direction and manuscript editing. SY supervised the study and provided science direction and manuscript editing. All authors contributed to the article and approved the submitted version.

## Funding

This research was supported by Japan Society for the Promotion of Science (JSPS) KAKENHI, Grant Number 18H04136 (SY).

## Conflict of interest

The authors declare that the research was conducted in the absence of any commercial or financial relationships that could be construed as a potential conflict of interest.

## Publisher’s note

All claims expressed in this article are solely those of the authors and do not necessarily represent those of their affiliated organizations, or those of the publisher, the editors and the reviewers. Any product that may be evaluated in this article, or claim that may be made by its manufacturer, is not guaranteed or endorsed by the publisher.

## Footnotes

^1^ https://qiime2.org/

^2^ http://hannonlab.cshl.edu/fastx_toolkit/index.html

^3^ http://cgenome.net/calypso/
