# Supplementary material for: Microbial communities on eelgrass (Zostera marina) thriving in Tokyo Bay and the possible source of leaf-attached microbes
Source: Front Microbiol. 2023 Jan 6;13:1102013. doi: 10.3389/fmicb.2022.1102013 (PMC9853538; doi:10.3389/fmicb.2022.1102013)
Supplement: Supplementary file 1 [file Data_Sheet_1.docx]

Supplementary Material

# Supplementary Figures and Tables

## Supplementary Figures

**Supplementary Figure 1.** Heatmap showing the order-level microbial community composition of the eelgrass phyllosphere (differently aged and decayed leaves), rooting zone (the root-rhizome) and surrounding environments (eelgrass surrounding water: T, PA, and FL fraction and rhizosphere sediment) collected in July and September. The x-axis displays samples clustered by similarity, and the y-axis displays bacterial orders. T: total (PA+FL); PA: particle-associated; FL: free-living; (L1-L3): differently aged leaves; L4: decayed leaves; RR: root-rhizome; RS: rhizosphere sediment.

**Supplementary Figure 2.** Alpha diversity of test samples. Two alpha diversity metrics, **(A)** Chao1 and **(B)** Shannon diversity indices, are shown as boxplots for the eelgrass phyllosphere (differently aged and decayed leaves), rooting zone (the root-rhizome), surrounding environments (eelgrass surrounding water and rhizosphere sediment), and control environment (bulk water and sediment). In the eelgrass phyllosphere, older leaves (i.e., L2 and L3) showed high microbial diversity compared with young leaves (i.e., L1), whereas decaying leaves (i.e., L4) showed the lowest diversity, except for Chao1 in L1. T: total (PA+FL); PA: particle-associated; FL: free-living; (L1-L3): differently aged leaves; L4: decayed leaves; RR: root-rhizome; RS: rhizosphere sediment; BS: bulk sediment.

**Supplementary Figure 3.** Boxplot showing the top 10 leaf bacteria of taxonomic abundance among leaf blades at the class level. The asterisks indicate statistically significant differences (*P < 0.001*). (L1-L3): differently aged leaves; L4: decayed leaves.

**Supplementary Figure 4.** UpSet plot showing the unique and shared sequence variants (ASVs) among the eelgrass phyllosphere (differently aged and decayed leaves) and suspended particles (PA). The 30 intersections that involve the greatest number of ASVs are displayed. PA: particle-associated; (L1-L3): differently aged leaves; L4: decayed leaves.

## Supplementary Table

**Supplementary Table 1.** Statistics of Illumina sequencing data and alpha diversity (Chao 1 and Shannon) values of each sample. T: total (PA+FL); PA: particle-associated; FL: free-living; (L1-L3): differently aged leaves; L4: decayed leaves; RR: root-rhizome; RS: rhizosphere sediment; BS: bulk sediment.

| **Sample type** | **Sample** | **replicate** | **Illumina sequencing data** | | | | | | **Alpha diversity** | |
| --- | --- | --- | --- | --- | --- | --- | --- | --- | --- | --- |
|  |  |  | **Before rarefied** | | | | **After rarefied** | | **Chao1** | **Shannon** |
|  |  |  | **Raw sequences** | **Nonchimeric** | **Filtered sequences** | **Number of ASVs** | **Filtered sequences** | **Number of ASVs** |  |  |
| Bulk water (July) | T | R1 | 133910 | 84202 | 81682 | 312 | 23000 | 311 | 313.00 | 4.68 |
|  |  | R2 | 112951 | 66893 | 65405 | 276 | 23000 | 276 | 276.00 | 4.55 |
|  |  | R3 | 121964 | 73551 | 72234 | 291 | 23000 | 290 | 290.00 | 4.58 |
|  | PA | R1 | 118120 | 74837 | 67691 | 276 | 23000 | 274 | 274.00 | 4.69 |
|  |  | R2 | 120971 | 69043 | 65144 | 309 | 23000 | 309 | 315.00 | 4.81 |
|  |  | R3 | 131584 | 80484 | 75046 | 326 | 23000 | 325 | 325.17 | 4.83 |
|  | FL | R1 | 117233 | 79512 | 76654 | 223 | 23000 | 222 | 225.00 | 4.41 |
|  |  | R2 | 121937 | 87300 | 86200 | 262 | 23000 | 262 | 262.43 | 4.54 |
|  |  | R3 | 119836 | 86217 | 84745 | 274 | 23000 | 274 | 275.20 | 4.57 |
| Eelgrass  surrounding water (July) | T | R1 | 125831 | 87458 | 84232 | 316 | 23000 | 315 | 316.67 | 4.61 |
|  |  | R2 | 138606 | 98263 | 95687 | 299 | 23000 | 299 | 303.67 | 4.46 |
|  |  | R3 | 135498 | 93495 | 89462 | 342 | 23000 | 339 | 340.15 | 4.72 |
|  | PA | R1 | 132132 | 83365 | 81920 | 430 | 23000 | 428 | 428.67 | 5.24 |
|  |  | R2 | 109697 | 72982 | 70236 | 352 | 23000 | 352 | 353.67 | 4.93 |
|  |  | R3 | 134248 | 84821 | 82446 | 385 | 23000 | 384 | 384.43 | 4.93 |
|  | FL | R1 | 125866 | 93049 | 92717 | 221 | 23000 | 220 | 220.00 | 4.22 |
|  |  | R2 | 132808 | 100172 | 99756 | 237 | 23000 | 237 | 237.11 | 4.23 |
|  |  | R3 | 100867 | 67183 | 67035 | 180 | 23000 | 180 | 180.00 | 4.08 |

*Cont. Supplementary Table 1*

| **Sample type** | **Sample** | **Replicate** | **Illumina sequencing data** | | | | | | **Alpha diversity** | |
| --- | --- | --- | --- | --- | --- | --- | --- | --- | --- | --- |
|  |  |  | **Before rarefied** | | | | **After rarefied** | | **Chao1** | **Shannon** |
|  |  |  | **Raw sequences** | **Nonchimeric** | **Filtered sequences** | **Number of ASVs** | **Filtered sequences** | **Number of ASVs** |  |  |
| Leaf  (July) | L1 | R1 | 108253 | 81332 | 57708 | 272 | 23000 | 270 | 270 | 4.72 |
|  |  | R2 | 100741 | 61332 | 5874 | 60 | ND | ND | ND | ND |
|  |  | R3 | 100698 | 73887 | 25853 | 173 | 23000 | 173 | 173 | 4.33 |
|  |  | R4 | 112697 | 86279 | 23094 | 128 | 23000 | 128 | 128 | 3.44 |
|  |  | R5 | 93608 | 72425 | 26882 | 163 | 23000 | 163 | 163 | 4.12 |
|  |  | R6 | 121091 | 90197 | 55841 | 317 | 23000 | 317 | 317.25 | 5.05 |
|  |  | R7 | 97298 | 70955 | 50682 | 329 | 23000 | 328 | 328.5 | 5.18 |
|  |  | R8 | 119419 | 90049 | 25704 | 149 | 23000 | 149 | 149 | 4.26 |
|  | L2 | R1 | 130787 | 100744 | 97817 | 285 | 23000 | 283 | 284.25 | 4.49 |
|  |  | R2 | 133939 | 103278 | 101948 | 267 | 23000 | 267 | 270.5 | 4.29 |
|  |  | R3 | 132156 | 101041 | 98125 | 299 | 23000 | 297 | 297.67 | 4.56 |
|  | L3 | R1 | 112620 | 81283 | 76701 | 321 | 23000 | 317 | 317.5 | 4.58 |
|  |  | R2 | 119280 | 85764 | 75745 | 338 | 23000 | 338 | 338.55 | 4.74 |
|  |  | R3 | 128529 | 91447 | 78856 | 366 | 23000 | 362 | 362.77 | 4.83 |
|  | L4 | R1 | 127710 | 95656 | 95617 | 81 | 23000 | 80 | 80 | 2.52 |
|  |  | R2 | 130287 | 91197 | 90616 | 358 | 23000 | 356 | 358.1 | 4.35 |
|  |  | R3 | 122276 | 90650 | 90368 | 177 | 23000 | 175 | 175 | 3.41 |
| Root-rhizome (July) | RR | R1 | 125280 | 86389 | 84154 | 470 | 23000 | 470 | 475.06 | 5.15 |
|  |  | R2 | 132516 | 98377 | 92879 | 436 | 23000 | 435 | 437.77 | 5.1 |
|  |  | R3 | 141569 | 81805 | 79097 | 570 | 23000 | 566 | 567.24 | 5.52 |
| Rhizosphere- sediment (July) | RS | R1 | 147476 | 66306 | 65854 | 455 | 23000 | 455 | 455.75 | 5.49 |
|  |  | R2 | 128305 | 55171 | 54609 | 415 | 23000 | 414 | 415.25 | 5.44 |
|  |  | R3 | 135573 | 55441 | 55051 | 403 | 23000 | 403 | 403.38 | 5.29 |
| Bulk sediment (July) | BS | R1 | 126471 | 50651 | 45841 | 413 | 23000 | 413 | 414.2 | 5.45 |
|  |  | R2 | 109837 | 47567 | 46800 | 691 | 23000 | 691 | 691.75 | 5.97 |
|  |  | R3 | 126231 | 52135 | 51529 | 421 | 23000 | 420 | 422.5 | 5.54 |

*Cont. Supplementary Table 1*

| **Sample type** | **Sample** | **replicate** | **Illumina sequencing data** | | | | | | **Alpha diversity** | |
| --- | --- | --- | --- | --- | --- | --- | --- | --- | --- | --- |
|  |  |  | **Before rarefied** | | | | **After rarefied** | | **Chao1** | **Shannon** |
|  |  |  | **Raw sequences** | **Nonchimeric** | **Filtered sequences** | **Number of ASVs** | **Filtered sequences** | **Number of ASVs** |  |  |
| Eelgrass  surrounding water (Sept.) | T | R1 | 133244 | 101149 | 96556 | 243 | 23000 | 240 | 244.5 | 4.19 |
|  |  | R2 | 122573 | 90867 | 84469 | 279 | 23000 | 276 | 280 | 4.31 |
|  |  | R3 | 115819 | 87347 | 80382 | 285 | 23000 | 282 | 283 | 4.4 |
|  | PA | R1 | 107397 | 77051 | 61058 | 315 | 23000 | 312 | 316.2 | 4.78 |
|  |  | R2 | 126007 | 90870 | 74831 | 376 | 23000 | 372 | 379.8 | 4.88 |
|  |  | R3 | 125211 | 87981 | 70505 | 357 | 23000 | 355 | 356.43 | 4.91 |
|  | FL | R1 | 119526 | 92759 | 92672 | 201 | 23000 | 197 | 199.63 | 4.12 |
|  |  | R2 | 117094 | 90730 | 90605 | 216 | 23000 | 213 | 215.5 | 4.16 |
|  |  | R3 | 120351 | 97076 | 97041 | 204 | 23000 | 201 | 201.6 | 3.94 |
| Leaf  (Sept.) | L1 | R1 | 111294 | 84007 | 51993 | 263 | 23000 | 261 | 262.43 | 4.48 |
|  |  | R2 | 103863 | 78931 | 40941 | 249 | 23000 | 249 | 249.17 | 4.65 |
|  |  | R3 | 102859 | 75759 | 41725 | 250 | 23000 | 250 | 250.25 | 4.51 |
|  | L2 | R1 | 130238 | 96810 | 68335 | 487 | 23000 | 486 | 486.6 | 5.47 |
|  |  | R2 | 118649 | 83368 | 50634 | 406 | 23000 | 406 | 406.3 | 5.27 |
|  |  | R3 | 122630 | 85002 | 54645 | 471 | 23000 | 468 | 471.11 | 5.42 |
|  | L3 | R1 | 117341 | 89359 | 75403 | 400 | 23000 | 394 | 396.77 | 5.04 |
|  |  | R2 | 105955 | 75836 | 61370 | 367 | 23000 | 365 | 366.88 | 5.03 |
|  |  | R3 | 107156 | 79119 | 49907 | 304 | 23000 | 302 | 302.38 | 4.76 |
|  | L4 | R1 | 113263 | 81879 | 81620 | 256 | 23000 | 254 | 256 | 4.19 |
|  |  | R2 | 115839 | 79479 | 79127 | 254 | 23000 | 252 | 254.8 | 4.25 |
|  |  | R3 | 113052 | 80825 | 80592 | 233 | 23000 | 232 | 233.2 | 3.99 |
| Root-rhizome (Sept.) | RR | R1 | 129320 | 86869 | 70013 | 452 | 23000 | 450 | 451.75 | 4.99 |
|  |  | R2 | 109533 | 70556 | 37736 | 356 | 23000 | 354 | 354 | 5.02 |
|  |  | R3 | 139641 | 90666 | 72214 | 600 | 23000 | 593 | 606.13 | 5.65 |
| Rhizosphere- sediment (Sept.) | RS | R1 | 114364 | 73592 | 66222 | 504 | 23000 | 501 | 503 | 5.51 |
|  |  | R2 | 128830 | 74316 | 70764 | 674 | 23000 | 662 | 668.84 | 5.79 |
|  |  | R3 | 121358 | 65104 | 61782 | 589 | 23000 | 583 | 589.67 | 5.73 |

‘ND’ Not determined
